# PLOD Family: A Novel Biomarker for Prognosis and Personalized Treatment in Soft Tissue Sarcoma

**DOI:** 10.3390/genes13050787

**Published:** 2022-04-28

**Authors:** Siming Gong, Nikolas Schopow, Yingjuan Duan, Changwu Wu, Sonja Kallendrusch, Georg Osterhoff

**Affiliations:** 1 Institute of Anatomy, University of Leipzig, Liebigstraße 13, 04103 Leipzig, Germany; siming.gong@studserv.uni-leipzig.de (S.G.); nikolas.schopow@medizin.uni-leipzig.de (N.S.); sonja.kallendrusch@medizin.uni-leipzig.de (S.K.); 2 Sarcoma Center, Department for Orthopedics, Trauma Surgery and Reconstructive Surgery, University Hospital Leipzig, Liebigstraße 20, 04103 Leipzig, Germany; georg.osterhoff@medizin.uni-leipzig.de; 3 Faculty of Chemistry and Mineralogy, University of Leipzig, Johannisallee 29, 04103 Leipzig, Germany; yingjuan.duan@studserv.uni-leipzig.de; 4 Department of Medicine, Health and Medical University Potsdam, Olympischer Weg 1, 14471 Potsdam, Germany

**Keywords:** PLOD family, soft tissue sarcoma, prognostic, tumor microenvironment, immune infiltration, personalized treatment

## Abstract

Despite various treatment attempts, the heterogenous group of soft tissue sarcomata (STS) with more than 100 subtypes still shows poor outcomes. Therefore, effective biomarkers for prognosis prediction and personalized treatment are of high importance. The Procollagen-Lysine, 2-Oxoglutarate 5-Dioxygenase (PLOD) gene family, which is related to multiple cancer entities, consists of three members which encode important enzymes for the formation of connective tissue. The relation to STS, however, has not yet been explored. In this study, data from The Cancer Genome Atlas (TCGA) and Genotype-Tissue Expression (GTEx) databases were used to analyze the role of PLOD1–3 in STS. It was found that an overexpression of PLOD family members correlates with poor prognosis, which might be due to an increased infiltration of immune-related cells in the tumor microenvironment. In STS, the expression of PLOD genes could be a novel biomarker for prognosis and a personalized, more aggressive treatment in these patients.

## 1. Introduction

Soft tissue sarcomata (STS) are a family of rare mesenchymal malignancies with more than 100 subtypes. Still, STS are responsible for 19–21% of all cancer-related deaths in childhood and adolescence [1,2,3,4]. Although various approaches such as surgical resection, radiotherapy, chemotherapy, and immunotherapy or dual or triple combinations have been applied to STS already, the outcome is still poor [5,6,7,8,9]. In the current guidelines of the American and European oncological societies, specific systemic treatments are only recommended for a few subtypes (e.g., rhabdomyosarcoma) [10,11]. Due to the divergence between many subtypes and a low incidence of sarcomata, there is a lack of substantial treatment data and evidence-based therapy concepts. In the past, most subtypes of STS have been grouped together and treated according to a “one-size-fits-all” method [12,13].

In recent years, however, there has been a trend towards a more precise and personalized management of STS [14,15,16,17]. Subtype-dependent treatments have already been able to improve the prognosis of uterine, rhabdomyo-, and fibromyxoid sarcomata [18,19,20,21]. In addition, some genes, such as *BCL-2* and *SHP-1*, have already been linked to STS, from which new promising therapeutic strategies could be identified [22,23,24,25,26,27]. A better understanding of the oncogenesis of STS is needed, as well as robust biomarkers to differentiate between subtypes and between patients with different risk profiles.

The extracellular matrix (ECM) is present in all kinds of tissues and gets in touch with various cells including the mesenchymal cells [28]. Collagen is one of the main components in ECM and it is crucial not only for normal cell function but also for tumor formation [28]. Abnormal collagen may result in various diseases, tumor formation, and metastases [29,30,31]. Eisinger-Mathason et al. suggested that the modification collagen may promote sarcomata metastasis [32]. Lysyl hydroxylase takes part in the process of covalent cross-links and collagen glycosylation and is catalyzed by Procollagen-Lysine, 2-Oxoglutarate 5-Dioxygenases (PLODs). In addition, the deposition and crosslink in the extracellular matrix provide the chemical and physical basement for cancer formation and proliferation [28]. There are three members in the PLOD family, called PLOD1, PLOD2, and PLOD3, and their overexpression may result in tumor progression [28]. 

With an overall identity in protein sequences of 47%, the members of the PLOD family are highly homologous [33]. Lysyl residues in the triple helix are hydroxylated by PLOD1 and PLOD3 and lysyl residues in the telopeptides of collagen are hydroxylated by PLOD2 [34]. PLOD1 is, among other things, essential for the formation of healthy bone tissue. It influences wound healing and vascular stability and has recently been linked to oncogenesis and metastasis of osteosarcomata (and, i.a., bladder cancer, gastric cancer, glioblastoma, and lung adenocarcinoma) [35,36,37,38,39,40,41,42,43]. PLOD2 is required for the permanent cross-linking of collagen in the extracellular matrix. Defects result in fibrosis, osteogenesis imperfecta, and Bruck syndrome [44,45,46]. PLOD2 plays a key role in cancer cell migration and invasion, e.g., of osteosarcomata (and, i.a., breast cancer, cervical cancer, colon cancer, renal cell carcinoma, squamous cell carcinoma, and lung cancer) [47,48,49,50,51,52,53,54,55,56]. It also affects the chemotherapy resistance of various tumor cell types [57,58]. The activity of PLOD3 is important for the biosynthesis of collagen type IV and VI, and our previous work has shown that PLOD3 is highly expressed in STS [59,60,61]. In addition, overexpression of PLOD3 could lead to poor prognosis in tumors such as gastric cancer, colon adenocarcinoma, and glioma [62,63,64,65]. Some studies have suggested that the PLOD family and its members could regulate the immune infiltration in some tumors such as gastric cancer, pancreatic cancer, lung adenocarcinoma, and low-grade glioma [30,66,67,68,69]. 

Previous studies also showed that the PLOD family could be promising biomarkers and a potential therapy target for a variety of carcinoma. Consequently, we speculated that the PLODs family members individually or in combination could also be potential biomarkers for prediction of prognosis and personalized management in STS. 

## 2. Materials and Methods

### 2.1. ONCOMINE Analysis

The expression of PLOD family members in various cancers at mRNA level was analyzed by the ONCOMINE tool (www.oncomine.org, accessed on 1 December 2021). In this study, we used the “Gene summary view” and “dataset view”. Differences in means were detected using Student’s *t*-test. The parameters used in this study were: *p* value < 0.05, Fold change > 2. The type of analysis: Cancers vs. normal; and the level of the data: mRNA.

### 2.2. Datasets

Xena (http://xena.ucsc.edu/, accessed on 1 December 2021) was used to obtain the RNA-sequencing (RNA-seq) data and clinical information of 263 STS samples from The Cancer Genome Atlas (TCGA) cohort and the RNA-seq data of 911 muscle or adipose samples from Genotype-Tissue Expression (GTEx) database. In total, 189 patients from TCGA with complete clinical data were included for final analysis (Table 1).

All data were normalized and log^2^ (x + 1) transformed. In addition, the batch effects were removed before the analysis of PLOD family members expression in STS was performed. 

### 2.3. Kaplan–Meier Plotter Analysis

The Kaplan–Meier Plotter (https://kmplot.com/analysis/, accessed on 1 December 2021) was used to analyze the survival data of PLOD family members in STS in TCGA and GEO. The samples were split into high-expression and low-expression groups with the best cut off by the expression of PLOD family members. The Kaplan–Meier survival curves were obtained, in which the *p* values were gained based on log-rank test. The hazard ratios (HRs) and *p* values were shown correspondingly.

### 2.4. Gene Enrichment Analysis

Gene expression profiling interactive analysis 2 (GEPIA2) (http://gepia2.cancer.pku.cn, accessed on 1 December 2021) was used to obtain the genes related to PLOD1–3. GeneMANIA (https://genemania.org/, accessed on 1 December 2021) was used to harvest the top 20 genes which are most related to PLOD family members (Supplementary Table S1). Based on the combination of the data from GEPIA2 and GenaMANIA, “clusterProfiler” R package was employed to perform the Kyoto Encyclopedia of Genes and Genomes (KEGG) pathway analysis and Gene Ontology (GO) enrichment analysis.

### 2.5. TIMER Analysis

The Tumor Immune Estimation Resource (TIMER) (https://cistrome.shinyapps.io/timer/, accessed on 1 December 2021) website tool provides exhaustive analyses for immune-related infiltration over a variety of cancers including STS. The TIMER tool was used in this study to analyze six different kinds of immune cells in STS (B cells, CD8+ and CD4+ T cells, macrophages, neutrophils, and dendritic cells).

### 2.6. Immune Infiltration Analysis

The R package “GSVR” was employed to perform the single sample gene set enrichment analysis (ssGSEA) to assess the correlative abundance of 28 immune cells [70]. In addition, the R package “ESTIMATE” was used to evaluate three scores including the ImmuneScore (positively related to the immune cells infiltrates degree in cancers), StromalScore (positively related to the stroma cells in cancers), and ESTIMATEScore (negatively related to cancers purity) [71]. To explore the possible molecular mechanism of PLOD family members in STS, the R software (Version 4.0.3, R Foundation for Statistical Computing, Vienna, Austria) was employed to obtain the Gene set enrichment analysis (GSEA) based on the HALLMARK gene set [72].

## 3. Results

### 3.1. The Expression of PLOD Family Members in STS

**Figure 1:**
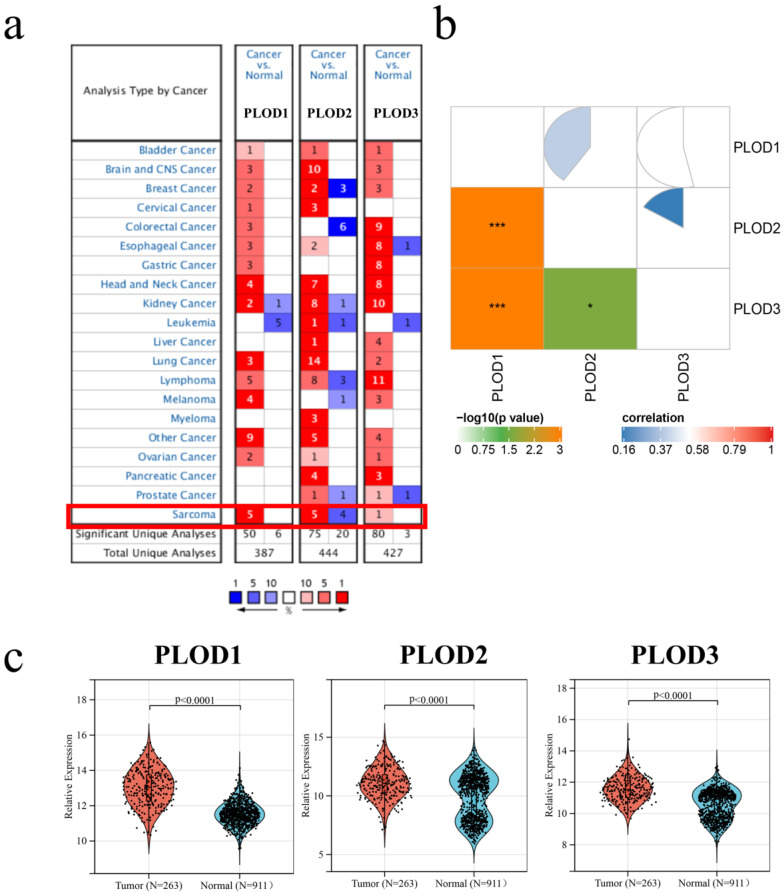
The expression of PLOD family members in STS using TCGA and GTEx datasets. (**a**) Expression in cancers compared to the corresponding normal tissue at transcription. The number in the cell states the amount of related analyses, which means in how many experiments the PLOD family members were high- or low- expression. The color of each cell depended upon the gene rank percentile. Red—up-/blue—downregulation. (**b**) Relation of expression levels among PLOD1–3 based on TCGA and GTEx cohort. (**c**) PLODs family members’ expressions between cancer tissues and corresponding normal tissues at transcriptional level. *: *p* < 0.05, ***: *p* < 0.001.

The STS data from the TCGA database and data from corresponding normal tissues from the GTEx cohort were analyzed. The comparison shows that PLOD1–3 were overexpressed in STS tumor tissues compared to the normal tissue (Figure 1c, *p* < 0.0001). In addition, the expression of PLOD family members is positively correlated with each other in STS (Figure 1b, all *p* < 0.05). The expression of PLOD family members was obtained based on ONCOMINE analysis. PLOD1 and PLOD3 were highly expressed in STS while PLOD2 was highly expressed in five studies but showed low expression in four studies (Figure 1a, *p* < 0.05), which means that PLOD2 was highly expressed in five experiments but showed low expression in four other experiments.

### 3.2. Survival Analysis Based on the Expression of PLOD Family Members

**Figure 2:**
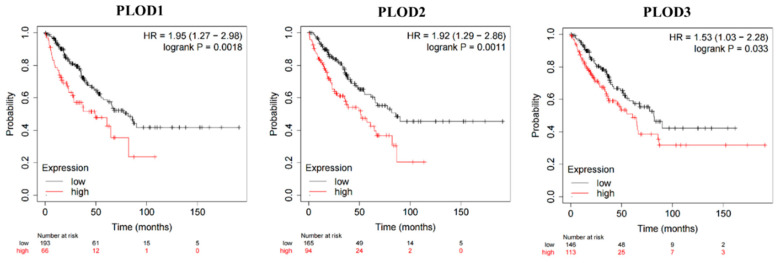
The survival analysis of expression of PLOD1–3 in STS. High expression of PLOD1, PLOD2, and PLOD3 were related with poor OS in STS.

The Kaplan–Meier Plotter tool was employed to explore the correlation of PLOD family members’ expression with the overall survival (OS) in STS. High expression of PLOD1–3 results in poor prognosis in STS (Figure 2, all *p* < 0.05). The hazard ratios range from 1.53 (PLOD3) to 1.95 (PLOD1).

### 3.3. The Expression of PLOD Family Members in Context of Age, Gender, and Grading

**Figure 3:**
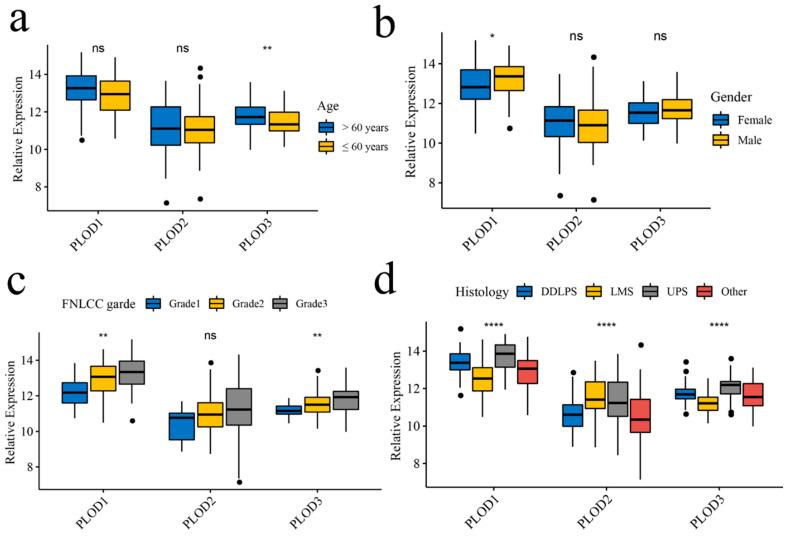
PLOD family members’ expressions in STS among age, gender, and grading/histology. (**a**) Age, (**b**) gender, (**c**) FNCLCC grades, (**d**) histology types. *: *p* < 0.05, **: *p* < 0.01, ****: *p* < 0.0001, ns: not significant.

The relation of expression of PLOD family members to various clinical parameters was analyzed based on the TCGA cohort. No significant differences were found in PLOD1 and PLOD2 among age groups but PLOD3 was highly expressed in patients older than 60 years (Figure 3a). The expression of PLOD2 and PLOD3 was not significantly correlated with the gender of patients, while PLOD1 was more highly expressed in male patients (Figure 3b). The stratified analysis shows that according to the Fédération Nationale des Centres de Lutte Contre le Cancer (FNCLCC), PLOD1 and PLOD3 are higher expressed in high FNCLCC grades compared with low grades of STS (Figure 3c). In addition, expression of all the PLOD family members is different in diverse histology types (Figure 3d).

### 3.4. KEGG Pathway and GO Enrichment Analyses for PLOD Family Members

**Figure 4:**
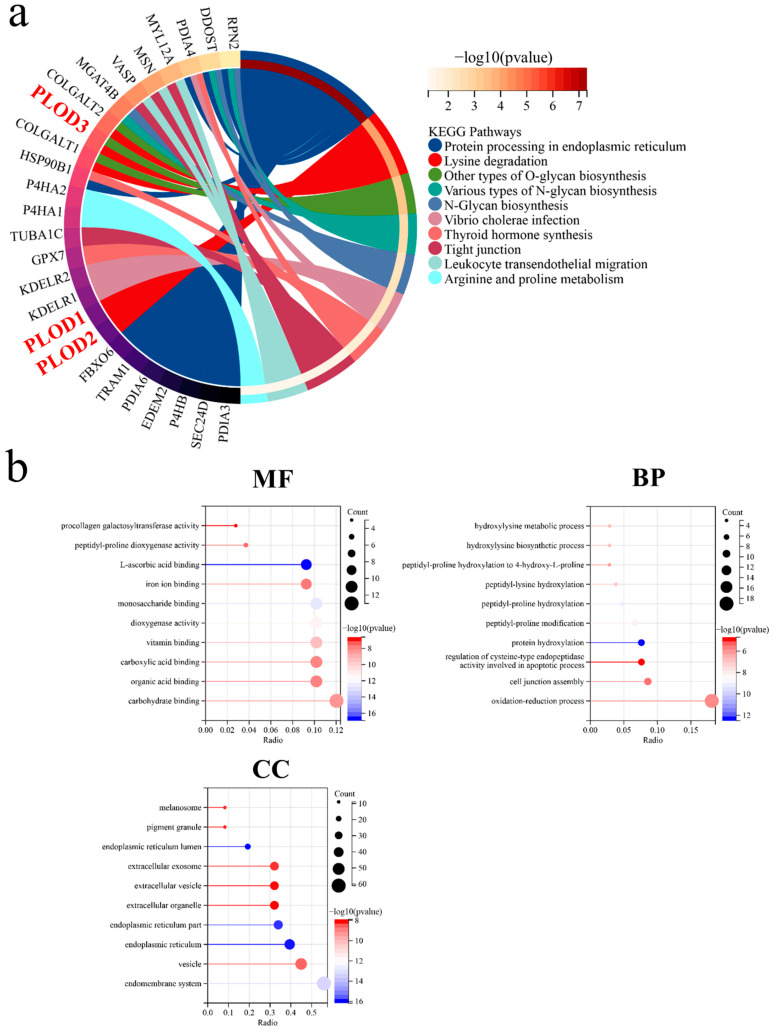
The PLOD family members’ genes enrichment analysis in STS. (**a**) KEGG pathway analysis and (**b**) GO enrichment analysis including molecular function (MF), biological process (BP), and cellular component (CC).

Based on the PLOD-related genes from GEPIA2 and Genemania, an enrichment analysis for PLOD1–3 was performed (Supplementary Figure S1 and Supplementary Table S1). According to the KEGG pathway analysis, PLOD1–3 were not only involved in protein processing in the endoplasmic reticulum (ER), but also in the immune-related pathways such as tight junction and leukocyte transendothelial migration (Figure 4a). The GO enrichment analysis indicated that PLOD family members are related to cell death-related pathways, such as the regulation of cysteine-type endopeptidase activity involved in the apoptotic process.

### 3.5. The Expression of PLOD Family Members with Tumor Immune Infiltration Cells

**Figure 5:**
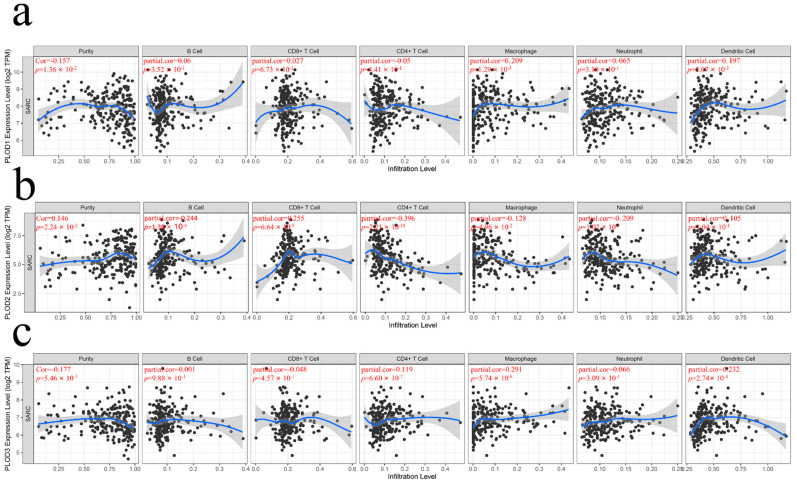
Correlations of PLOD family members’ expression with immune cells infiltration level in STS. The correlation between expression of (**a**) PLOD1, (**b**) PLOD2, (**c**) PLOD3, and each type of tumor immune infiltration cells.

To explore immune cell infiltration in TME in STS, the correlation of the PLOD family members’ expression to six immune cells was analyzed. PLOD1 is positively related to macrophage and dendritic cell infiltration (Figure 5a, both *p* < 0.05). PLOD2 is positively related to B cells and CD8+ T cells infiltration while negatively related to CD4+ T cells, macrophages, and neutrophils (Figure 5b, all *p* < 0.05). PLOD3 is positively related to CD4+ T cells, macrophages, and dendritic cells infiltration (Figure 5c, all *p* < 0.05).

### 3.6. Cellular and Molecular Characteristics of PLOD Family Members

**Figure 6:**
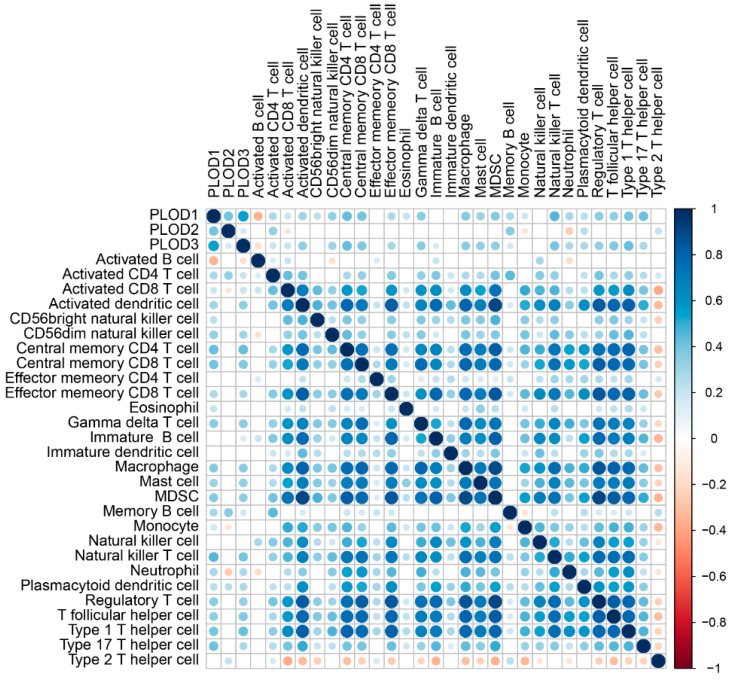
The immune landscape of PLOD family members and 28 immune cell types in STS.

**Figure 7:**
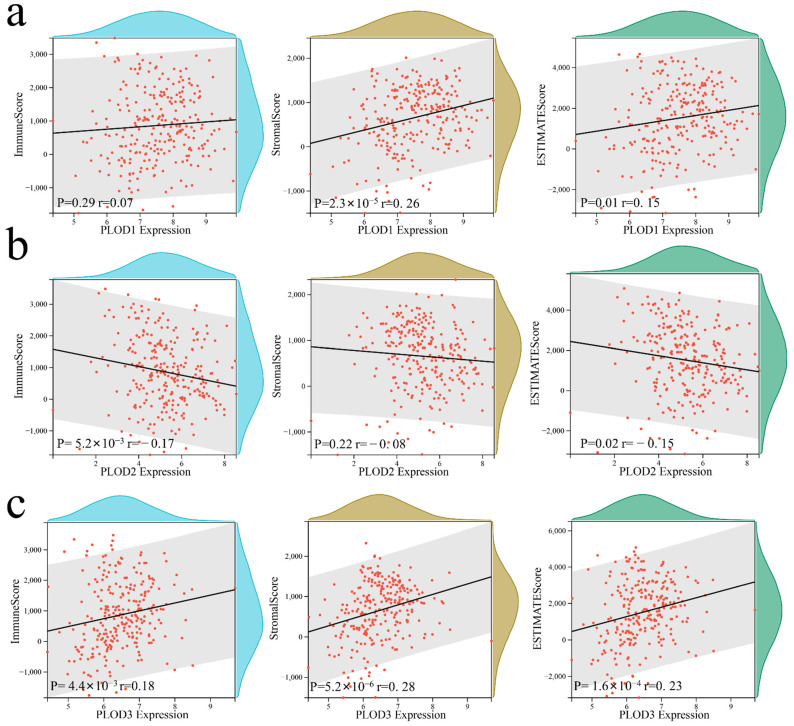
The overview of PLOD family members’ correlation with ImmuneScore, StromaScore, and ESTIMATEScore. (**a**) PLOD1, (**b**) PLOD2, and (**c**) PLOD3.

PLOD1 and PLOD3 are positively correlated with most of the immune cells while the PLOD2 are positively correlated with activated CD4+ T cells, memory B cells, plasmacytoid dendritic cells, and type 2 T helper cells infiltration level (Figure 6, all *p* < 0.05). This implies that a high expression of PLOD family members is linked to both an immune-stimulated and an immune-suppressed status, which indicated that the PLOD family could quantify the TME pattern of individual patients. It was found that the ImmuneScore is positively related to PLOD3 but negatively related to PLOD2, the StromaScore is positively related to PLOD1 and PLOD3, and the ESTIMATEScore is positively related to PLOD1 and PLOD3 but negatively related to PLOD2 (Figure 7, all *p* < 0.05).

### 3.7. Gene Set Enrichment Analysis of PLOD Family Members

**Figure 8:**
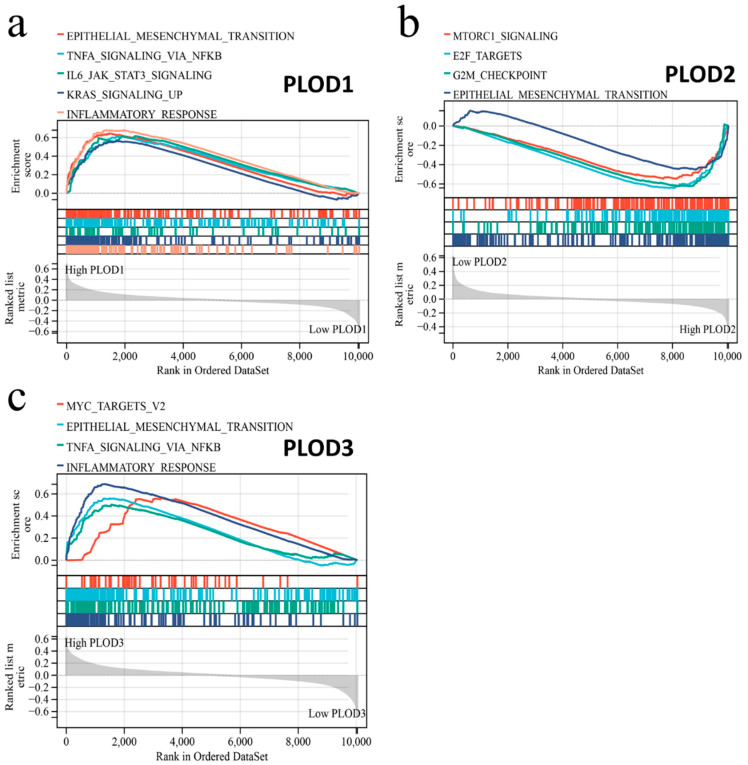
Gene set enrichment analysis of PLOD family members. The relation of PLODs to biological processes and signal transduction pathways using the HALLMARK gene set. (**a**) PLOD1, (**b**) PLOD2, and (**c**) PLOD3.

Gene set enrichment analysis (GSEA) was employed to obtain the possible biological processes and signal transduction pathways which were related to PLOD family members. It indicated that a high expression of PLOD family members was related to epithelial–mesenchymal transition and TNFA signaling (Figure 8 and Supplementary File S1).

## 4. Discussion

Jiang et al. suggested that PLOD1 could be a potential biomarker for the prognosis in osteosarcomata [38]. Additionally, PLOD3 showed overexpression and a correlation to prognosis in various kinds of tumors including STS based on bioinfomatic analysis and experiment validation [32]. Further studies have shown that the PLOD family members may be a possible biomarker for a wide range of cancers. This study sought to systematically investigate a potential correlation between the three PLOD family members and STS based on public available datasets (TCGA and GTEx).

PLOD1–3 catalyze the lysyl hydroxylase which is involved in the process of collagen formation [28]. Hence, mutations of PLOD family members could result in the disorder of connective tissues, such as Ehlers–Danlos and Bruck syndromes [73]. Epithelial–mesenchymal transition (EMT) is a procedure in which epithelial cells may obtain features from mesenchymal tissue, which means that the epithelial cells become unstable and lose their capacity of adherence [74]. The EMT is a common process in wound healing and tissue repair. However, when the epithelial cells lose their adherence ability and become unstable, tumors arise or even metastasize [75]. The overexpression of PLODs during connective tissue disorder and repair and EMT may occur, resulting in tumor formation. The GSEA analysis of the present study supports the hypothesis that PLOD family members were associated with EMT. PLOD1–3 and related genes were linked to protein biosynthesis and metabolic processes such as protein hydroxylation and hydroxylysine biosynthetic formation. However, there might be other mechanisms that could lead to tumor malignancy. In KEGG and GO analyses, PLOD1–3 were related to the endoplasmic reticulum (ER) and endomembrane system. Previous studies suggested that the mutation or different expression of some genes could induce abnormal function of the ER and further result in improperly folded proteins in soft tissues [76]. This prevents the ER from maintaining the homeostasis inside the cells by clearing out the misfolded or unfolded proteins [77]. The PLOD1 may be associated with ER stress in human Ehlers–Danlos syndrome [78]. PLOD3 was also linked to ER stress in human lung cancer models and mice models [79,80,81]. Thus, it might suggest that the ER stress could be a potential pathway in which the PLOD family could be used as the therapy target for STS. Interestingly, an experiment in mice suggested that radiation therapy is more effective while blocking ER stress-induced autophagy in sarcoma [82]. The findings of Marianne et al. suggested that sarcoma with low levels of ER stress was not sensitive to a proteasome inhibitor (bortezomib) [83]. Ritonavir could induce ER stress in bortezomib-resistant sarcoma cells, trigger the unfolded protein response, and maintain the high level of newly synthesized protein, but it would not block proteasomal active sites when compared to bortezomib. Consequently, although the single use of ritonavir or bortezomib would not influence tumor cell apoptosis, the combination of them could significantly increase the ER stress and then lead to >90% apoptosis [83], as the PLOD family is also associated with ER stress. Therefore, the PLOD family is not only suggested as promising biomarker but also as potential target for STS treatment.

TME plays a crucial role in tumor formation and even the response to treatment [84]. The TME contains several cell populations, including immune cells, endothelial cells, and fibroblasts [85]. Previous study indicated that the TME may have different characteristics in different types of tumors [86]. Consequently, the landscape of TME has become important for target-treatment and even the design of personalized therapy [86]. In this study, a comprehensive analysis of the infiltration of immune and stromal cells in TME was performed. The KEGG pathway analysis suggested that PLOD1–3 expression is linked to leukocyte trans-endothelial migration, whereas PLOD1 and PLOD3 are positively correlated with immunostimulatory and immunosuppressive cells while PLOD2 is positively correlated with activated CD4 T cells and plasmacytoid dendritic cells. These findings indicate that the tumors with a high expression of PLOD family members are in a state of immune “hot”, but immune-suppressed. This suggests that the expression of PLOD family members can indicate the TME pattern and might become helpful to set up an individual approach for personalized treatment.

PLOD family members were involved in various pathways of the tumor as well as the TME, which are linked to many subtypes of sarcoma. Consequently, the PLOD family members might be not only a potential marker, but also a potential target for an individual treatment design. Additionally, in recently years, the exosomes containing micro-RNA have become well-known in basic and clinical trials [87,88,89]. The exosomes containing PLOD family micro-RNA could be a promising method for the treatment of STS patients.

Taken together, the present study shows the potential role of PLOD1–3 in STS prognosis and their involvement in the immune-related infiltration in STS. It indicates that the PLOD family members could be viewed as biomarkers for the prognosis of STS as well as potential biomarkers for personalized treatment in STS.

## Figures and Tables

**Table 1:** Clinical characteristics of included STS patients.

Characteristics	All (*N* = 189)
Age group (Median)	
Younger (≤60 years)	96 (50.79%)
Older (>60 years)	93 (49.21%)
Gender	
Male	87 (46.03%)
Female	102 (53.97%)
Pathologic tumor size	
≤10.5 cm	95 (50.26%)
>10.5 cm	94 (49.74%)
Radiotherapy	
Yes	54 (28.57%)
No	135 (71.43%)
Pharmaceutical therapy	
Yes	45 (23.81%)
No	144 (76.19%)
FNCLCC grade	
1/2	115 (60.85%)
3	74 (39.15%)
Vital status	
Alive	117 (61.90%)
Dead	72 (38.10%)
Histological type	
DDLPS	49 (25.93%)
LMS	68 (35.98%)
UPS	41 (21.69%)
MFS	17 (8.99%)
SS	10 (5.29%)
MPNST	4 (2.12%)

LPS: dedifferentiated liposarcoma; LMS: leiomyosarcoma; UPS: undifferentiated pleomorphic sarcoma; MFS: myxofibrosarcoma; SS: synovial sarcoma; MPNST: malignant peripheral nerve sheath tumor.

## Data Availability

The data provided in this study can be obtained in the Methods section of this manuscript. The results shown here are, in part, based upon data generated by TCGA Research Network (https://www.cancer.gov/tcga, accessed on 1 December 2021), GTEx database (https://commonfund.nih.gov/gtex, accessed on 1 December 2021).

## Supplementary Materials

The following supporting information can be downloaded at: https://www.mdpi.com/article/10.3390/genes13050787/s1. Figure S1: Gene-gene interaction network among PLOD family members; Table S1: PLODs-correlated gene; File S1: Supplementary Files S1.
