# Supplementary figures and images for: PLOD Family: A Novel Biomarker for Prognosis and Personalized Treatment in Soft Tissue Sarcoma
Source: Genes (Basel). 2022 Apr 28;13(5):787. doi: 10.3390/genes13050787 (PMC9141206; doi:10.3390/genes13050787)

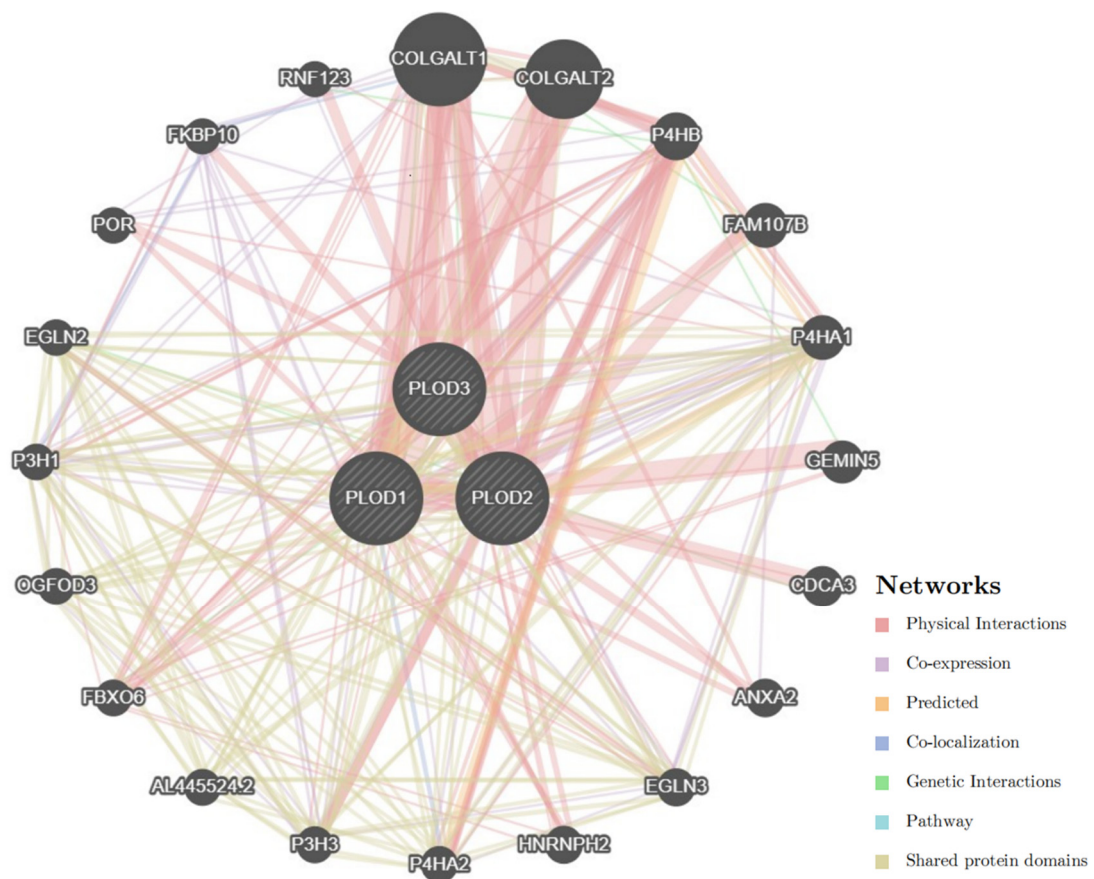

**Figure S1.** Gene-gene interaction network among PLOD family members. Each node represents a gene.

Supplement: Supplementary file 1 [file genes-13-00787-s001.zip › Supplementary Figure S1.pdf]
